# The T1 Hemorrhage Exclusion Sign in the Detection of Prostate Cancer at MRI

**DOI:** 10.5334/jbr-btr.1273

**Published:** 2017-03-29

**Authors:** Filippo Pesapane, Geert Villeirs, Pieter De Visschere

**Affiliations:** 1 Università degli Studi di Milano, IT; 2 Ghent University Hospital, De Pintelaan 185, Ghent, BE

**Keywords:** Biopsy, Magnetic resonance imaging (MRI), Prostate cancer, T1-weighted imaging

## Case History

A 76-year-old man, with no family history of prostate cancer (PCa), consulted for erectile dysfunction. Digital rectal examination revealed an enlarged prostate with a firm area on the left side. The serum PSA concentration was 17.70 ng/mL. Transrectal ultrasound (TRUS) examination of the prostate showed a hypoechoic area in the left peripheral zone (PZ). TRUS-guided biopsy revealed a Gleason score 9 (4 + 5) adenocarcinoma on the left side and a Gleason score 7 (4 + 3) adenocarcinoma on the right side.

For staging purposes, a 3T multiparametric MRI (mpMRI) was performed three weeks after the TRUS-guided biopsy. MRI confirmed a tumor in the midgland anterior and lateral posterior section of the left PZ with extracapsular extension and enlarged iliac lymph nodes.

**Figure 1:**
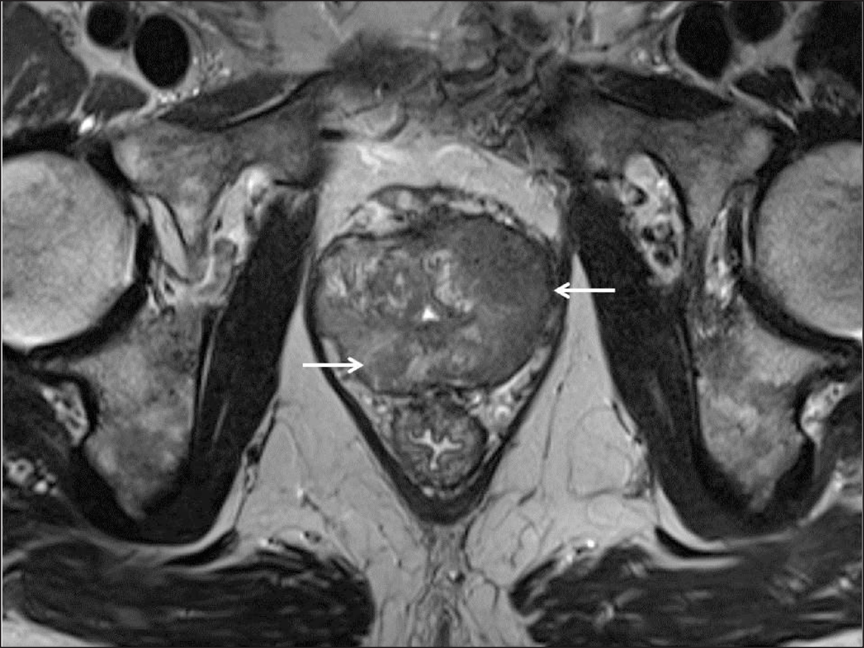
Axial T2WI. PCa lesions have homogeneous low signal intensity (arrows).

**Figure 2:**
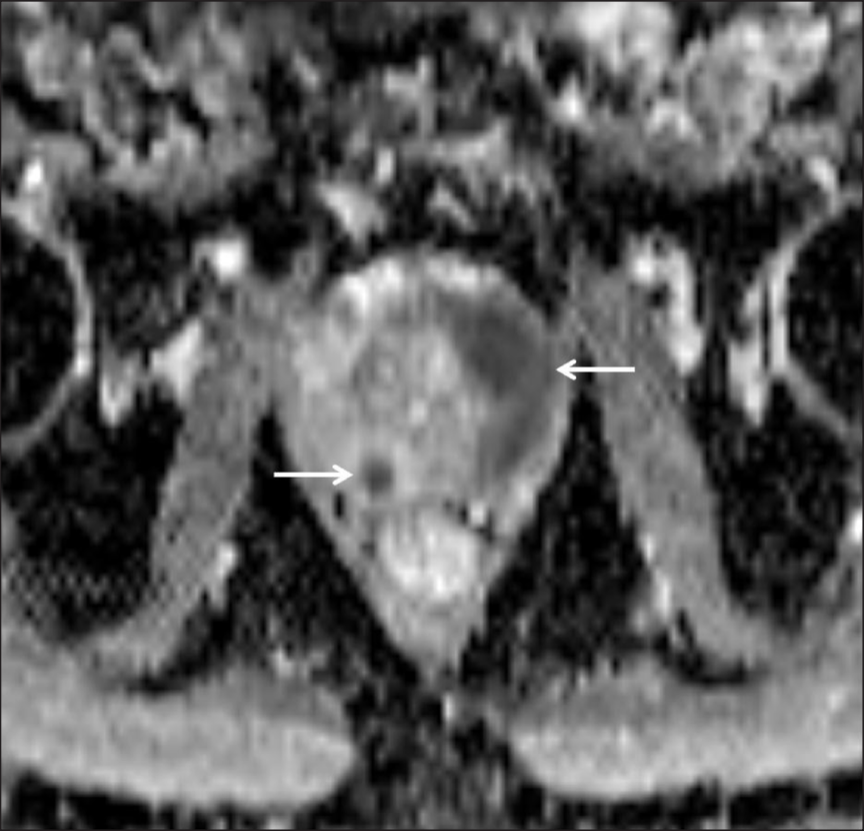
Axial ADC. PCa lesions appear markedly hypointense on ADC maps (arrows).

**Figure 3:**
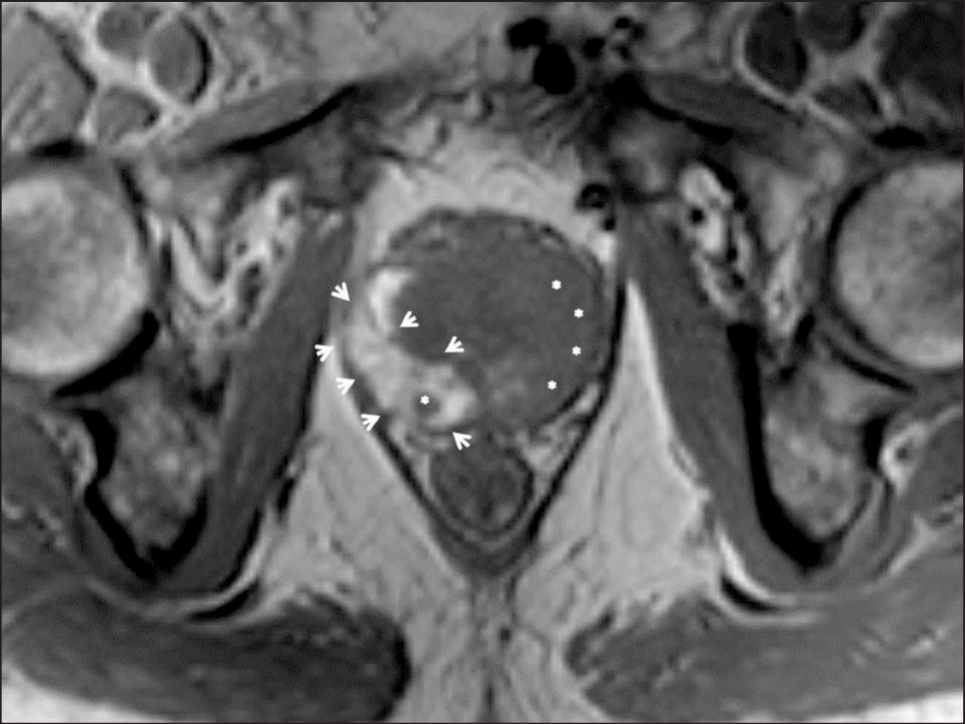
Axial T1. Diffuse hyperintense signal due to the post biopsy hemorrhage in the right PZ (arrowheads) with area free of T1 hyperintense blood (asterisks) matched the PCa lesions (T1 hemorrhage exclusion sign).

A second smaller nodular lesion was present in the midgland medial posterior section of the right PZ. The lesions had homogeneous low signal intensity on T2-weighted images (WI) (arrows on Figure 1) and low ADC value on the diffusion-weighted images (arrows on Figure 2). Axial T1–WI showed diffuse hyperintense signal due to the post biopsy hemorrhage in the right PZ, whereas the area free of T1 hyperintense blood remarkably matched the cancer localization (Figure 3: arrowheads delimit the area of hemorrhage, and asterisks indicate the tumors). This phenomenon has been described in the literature as the “T1 hemorrhage exclusion sign” [1].

## Comment

Intraprostatic hemorrhage is the most common biopsy-related morphological change. It can be identified as high signal intensity on T1–WI and can compromise the interpretation on T2–WI because of its similar appearance to that of PCa (low T2 signal intensity) in up to 80 percent of cases. However, the degree of hemorrhage has been shown to be significantly less in areas of PCa than in areas of normal or benign prostatic tissue. This feature has been referred to as the T1 hemorrhage exclusion sign. Although the prevalence of this sign is relatively low (20%), its positive predictive value for PCa is high (95%) and, as such, provides a useful diagnostic adjunct to DWI.

Some authors recommend delaying mpMRI for three to eight weeks after biopsy, but there is a large variability in the timing of the disappearance of hemorrhage. Furthermore, if the mpMRI is performed following a negative biopsy, the likelihood of PCa at the site of hemorrhage along a needle trajectory is low. In this situation, a clinically significant PCa should rather be expected in the non-hemorrhagic (non-biopsied) areas.

## Conclusion

The T1 hemorrhage exclusion sign is an additional aid for localization of larger foci of prostatic cancer on mpMRI performed after biopsy.
